# Simulation-based prediction of bone healing and treatment recommendations for lower leg fractures: Effects of motion, weight-bearing and fibular mechanics

**DOI:** 10.3389/fbioe.2023.1067845

**Published:** 2023-02-20

**Authors:** Marcel Orth, Bergita Ganse, Annchristin Andres, Kerstin Wickert, Elke Warmerdam, Max Müller, Stefan Diebels, Michael Roland, Tim Pohlemann

**Affiliations:** ^1^ Department of Trauma, Hand and Reconstructive Surgery, Saarland University, Saarbrücken, Germany; ^2^ Werner Siemens Endowed Chair of Innovative Implant Development (Fracture Healing), Saarland University, Saarbrücken, Germany; ^3^ Chair of Applied Mechanics, Saarland University, Saarbrücken, Germany

**Keywords:** biomechanics, motion, gait analysis, prognosis of bone healing, tibial fracture, lower leg injury, fibula, finite element analysis

## Abstract

Despite recent experimental and clinical progress in the treatment of tibial and fibular fractures, in clinical practice rates of delayed bone healing and non-union remain high. The aim of this study was to simulate and compare different mechanical conditions after lower leg fractures to assess the effects of postoperative motion, weight-bearing restrictions and fibular mechanics on the strain distribution and the clinical course. Based on the computed tomography (CT) data set of a real clinical case with a distal diaphyseal tibial fracture, a proximal and a distal fibular fracture, finite element simulations were run. Early postoperative motion data, recorded *via* an inertial measuring unit system and pressure insoles were recorded and processed to study strain. The simulations were used to compute interfragmentary strain and the von Mises stress distribution of the intramedullary nail for different treatments of the fibula, as well as several walking velocities (1.0 km/h; 1.5 km/h; 2.0 km/h) and levels of weight-bearing restriction. The simulation of the real treatment was compared to the clinical course. The results show that a high postoperative walking speed was associated with higher loads in the fracture zone. In addition, a larger number of areas in the fracture gap with forces that exceeded beneficial mechanical properties longer was observed. Moreover, the simulations showed that surgical treatment of the distal fibular fracture had an impact on the healing course, whereas the proximal fibular fracture barely mattered. Weight-bearing restrictions were beneficial in reducing excessive mechanical conditions, while it is known that it is difficult for patients to adhere to partial weight-bearing recommendations. In conclusion, it is likely that motion, weight bearing and fibular mechanics influence the biomechanical milieu in the fracture gap. Simulations may improve decisions on the choice and location of surgical implants, as well as give recommendations for loading in the postoperative course of the individual patient.

## 1 Introduction

Despite recent experimental and clinical progress in the treatment of tibial and fibular fractures, the rates of delayed bone healing and non-union remain challengingly high (Dailey et al., 2018). To date, patients with fractures of the lower leg can be treated by a variety of surgical techniques. During the postoperative course, they are often advised for restricted weight-bearing of the injured leg and are commonly followed up by regular clinical investigations and radiographic controls after at least after 6 and 12 weeks.

Although the etiology of disturbed bone healing may often be multifactorial, the mechanical environment within and around the fracture gap is known to be of crucial importance for the bone healing process (Claes and Cunningham, 2009). Accordingly, the objective measurement of mechanobiological parameters helps to determine progress of the fracture healing process. Biomechanical modification, e.g., *via* implants may lead to a different clinical outcome (Claes and Cunningham, 2009). In fact, previous studies identified the mechanical parameters ‘interfragmentary strain’ and ‘hydrostatic pressure’, and their threshold values as reliable parameters to monitor the different types of bone healing such as, e.g., intramembranous and endochondral ossification (Claes and Heigele, 1999; Shefelbine et al., 2005). Strain quantities beneficial or at least not harmful for bone healing have been identified in simulations and experiments, such as, e.g., octahedral shear strain and hydrostatic strain (Shefelbine et al., 2005), distortional strain (Simon et al., 2011; Ren and Dailey, 2020) or deviatoric strain (Son et al., 2014). However, the translation of these mostly experimental or simulation-based parameters to real human patient clinical cases remains an absolute rarity.

We have previously developed a simulation-based proof-of-concept workflow to determine the mechanical fracture environment after tibial fractures *in silico* (Braun et al., 2021). The aim of the present study was to simulate and compare different mechanical conditions after lower leg fractures, using individual postoperative motion data, to assess the effects of postoperative motion, weight-bearing restrictions and fibular biomechanics on the bone healing process of the tibia. For this purpose, a specific load case with a proximal and a distal fibular fracture in combination with a tibial injury was chosen for further analyses, because this fracture configuration allowed for concomitant comparisons of multiple tibio-fibular injuries. By these means, we aimed to find an individualized, optimal surgical and postoperative treatment recommendation.

## 2 Materials and methods

Ethical approval was obtained from the IRB of Saarland Medical Board (Aerztekammer des Saarlandes, Germany, application number 30/21). Informed consent was conducted according to the Declaration of Helsinki. The study is part of the project Smart Implants 2.0 – Weight-bearing and Gait Observation for Early Monitoring of Fracture Healing and Individualized Therapy after Trauma, funded by the Werner Siemens Foundation. It is registered in the German Clinical Trials Register (DRKS-ID: DRKS00025108).

### 2.1 Case data

**FIGURE 1:**
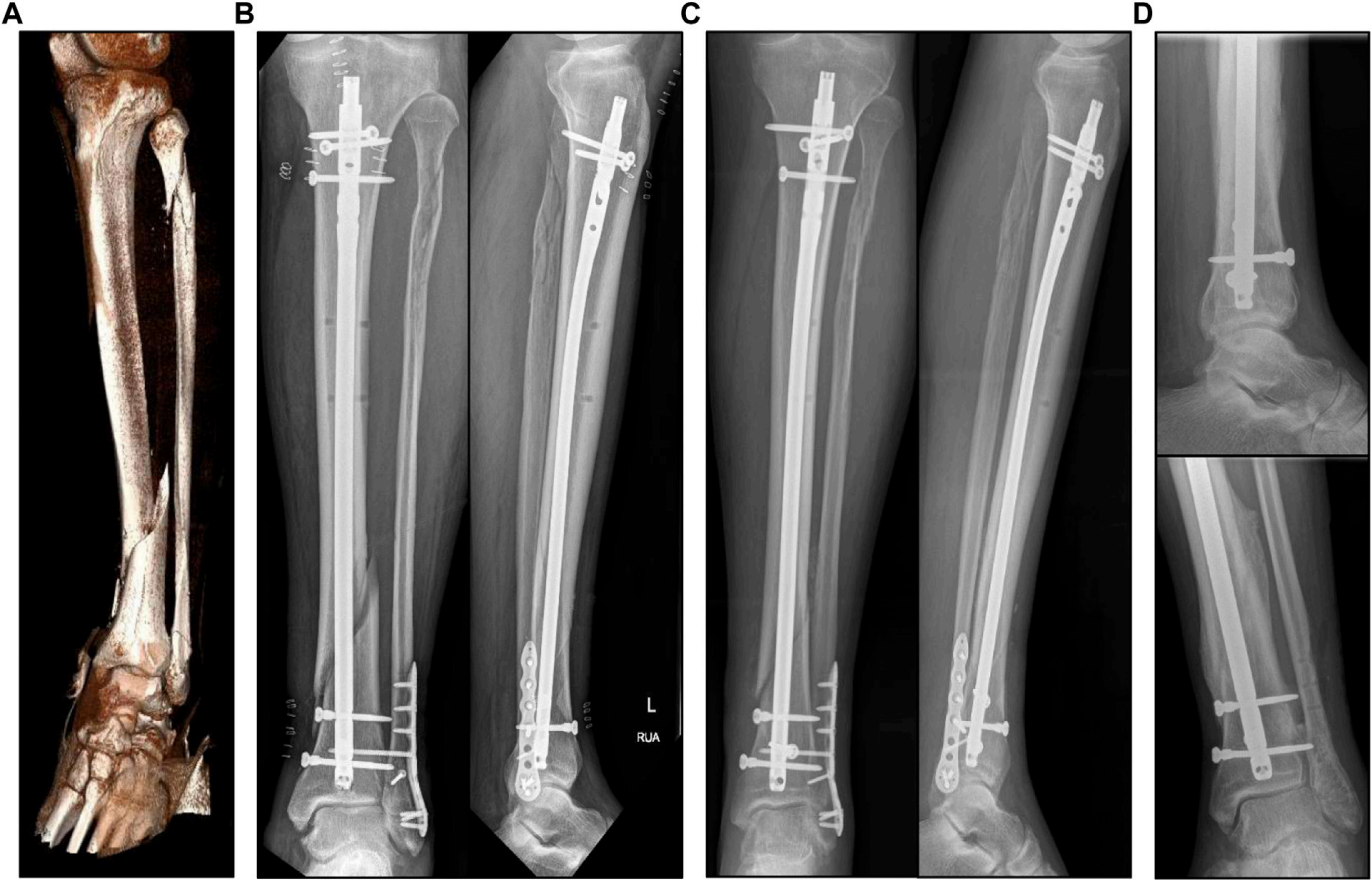
**(A)**: 3D reconstruction of the injured lower leg showing diaphyseal distal tibia fracture and a proximal and distal fibula fracture. **(B)**: X-rays antero-posterior and lateral view after surgical intervention by intramedullary nailing and plate osteosynthesis of the distal fibula fracture. **(C)**: X-ray control 6 weeks after surgery. **(D)**: Final X-ray approximately 7 months after surgery and shortly after plate removal at the distal fibula as well as removal of the set screw in the meantime. Note full osseous healing of the distal tibia and fibula fracture.

A 63-year-old male patient (height 180cm, weight 95 kg) suffered from a closed fracture of the lower leg with a distal diaphyseal fracture of the tibia, a proximal and a distal fibular fracture (Figure 1A). Computed tomography (CT) scans of the injured lower leg and the ankle joint were taken upon admission, and immediate Damage Control surgery was conducted on the day of the accident by closed reduction and the application of an ankle-joint-crossing, external fixator overspanning the fracture gap. After consolidation of the soft-tissue injury, the tibial fracture was surgically treated by implantation of an intramedullary nail (9 × 345 mm, Expert, Synthes, Umkirch, Germany). The distal fibular fracture was treated by open reduction and plate osteosynthesis (VariAx 2 Distal fibula system, Stryker, Kalamazoo, USA) including restoration of a syndesmotic injury by using a set screw, whereas the proximal fibular fracture was not treated surgically (Figure 1B). Postoperatively, the patient was mobilized on forearm crutches with a partial weight-bearing recommendation of 20 kg for the first 6 weeks (Figure 1C). A postoperative CT scan early after surgery and follow-up radiographs at 6 weeks and approximately 6 months after surgery were taken.

### 2.2 Computer modelling

To simulate different mechanical conditions in the fracture gap, a simulation workflow was applied, as previously described in detail (Braun et al., 2021). Briefly, the individual DICOM image stack of the patient’s postoperative CT scan was used to create geometric models. For this purpose, the images were segmented into masks by using an adaptive thresholding procedure. For each segmented mask, the morphological filters ‘island removal’, ‘cavity fill’ and ‘fill gaps’ were applied with a priority order resulting in high-quality segmentation. The results of the segmentation of the fracture gap were under visual control of the treating trauma surgeon and corrected, if necessary. After segmentation was completed, high-resolution adaptive finite element (FE) meshes were created using the software ScanIP (ScanIP, Synopsys, Mountain View, United States). Furthermore, material parameters were included into the FE meshes. The material parameter assignment for the masks of the intramedullary nail, the screws and the plate, as well as the fracture gap were chosen as homogeneous materials with standard properties derived from the literature (Imam and Fraker, 1996; Claes and Heigele, 1999). To assign the relationship between elasticity and bone density, the grayscale values of the CT data were mapped to the Hounsfield scale and to mechanical local bone properties (Hvid et al., 1989; Rho et al., 1995; Zannoni et al., 1998; Cattaneo et al., 2001). In line with previous studies, an isotropic heterogeneous material was assumed with a varying value for Young’s modulus and a fixed value for the Poisson ratio (Yosibash et al., 2007; Trabelsi et al., 2009). Depending on the local ash-density and the equivalent mineral density, the mapping for the cortical and the trabecular bone was defined as described elsewhere (Edwards et al., 2013; Knowles et al., 2016; Braun et al., 2021). All material properties were passed to the FE meshes and stored in the nodes and elements of the corresponding masks.

**FIGURE 2:**
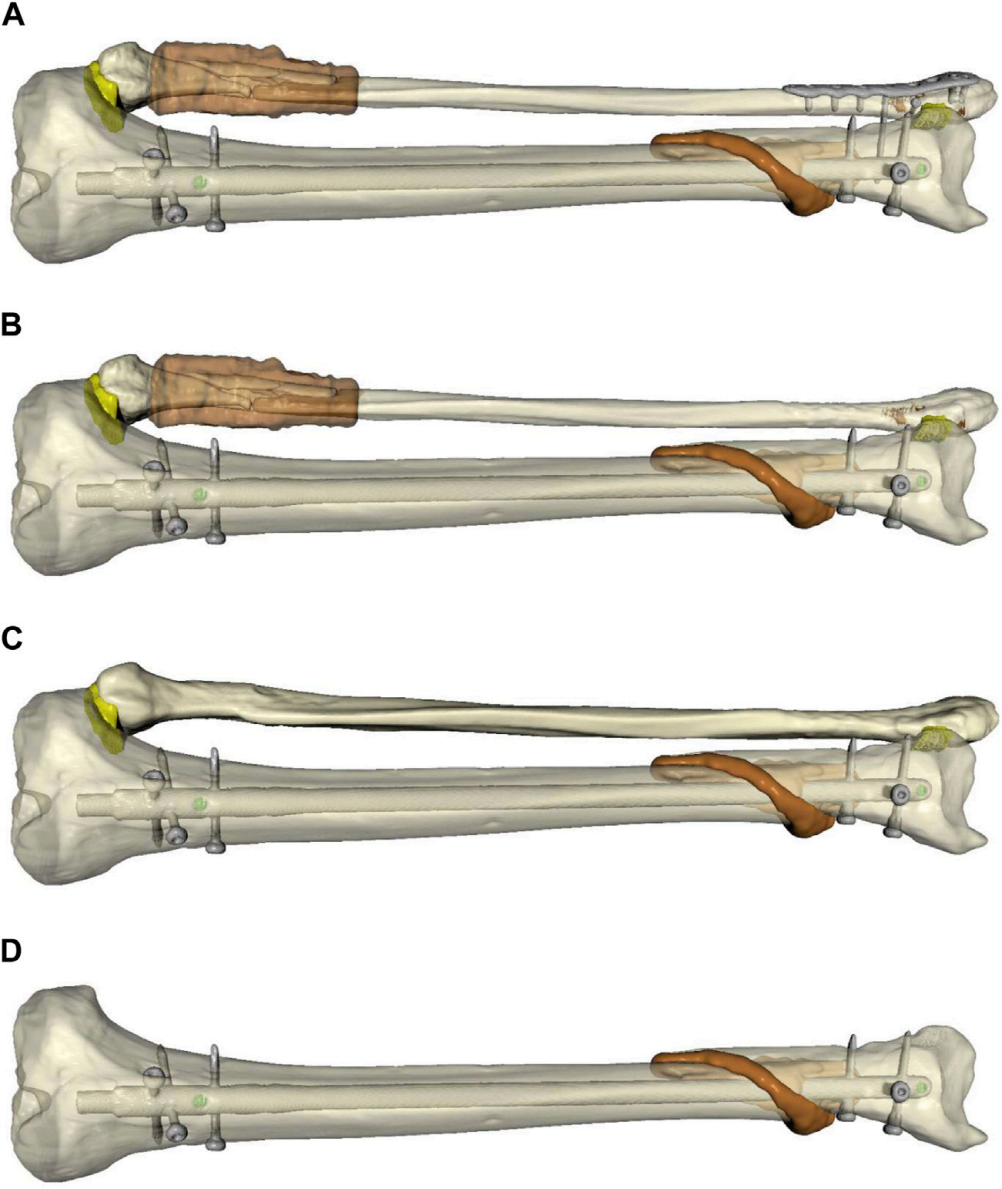
Simulated models of the individualized patient’s lower leg to compare different forms of biomechanical stability and surgical treatment options. **(A)**: Plate osteosynthesis of the distal fibular fracture and intramedullary nailing of the tibia, which resembles the real treatment of the patient (FibOP). **(B)**: Untreated fibula (Fib#). **(C)**: Intact fibula (Fib). **(D)**: Removed fibula, only simulation of the treated fibula (NoFib).

**FIGURE 3:**
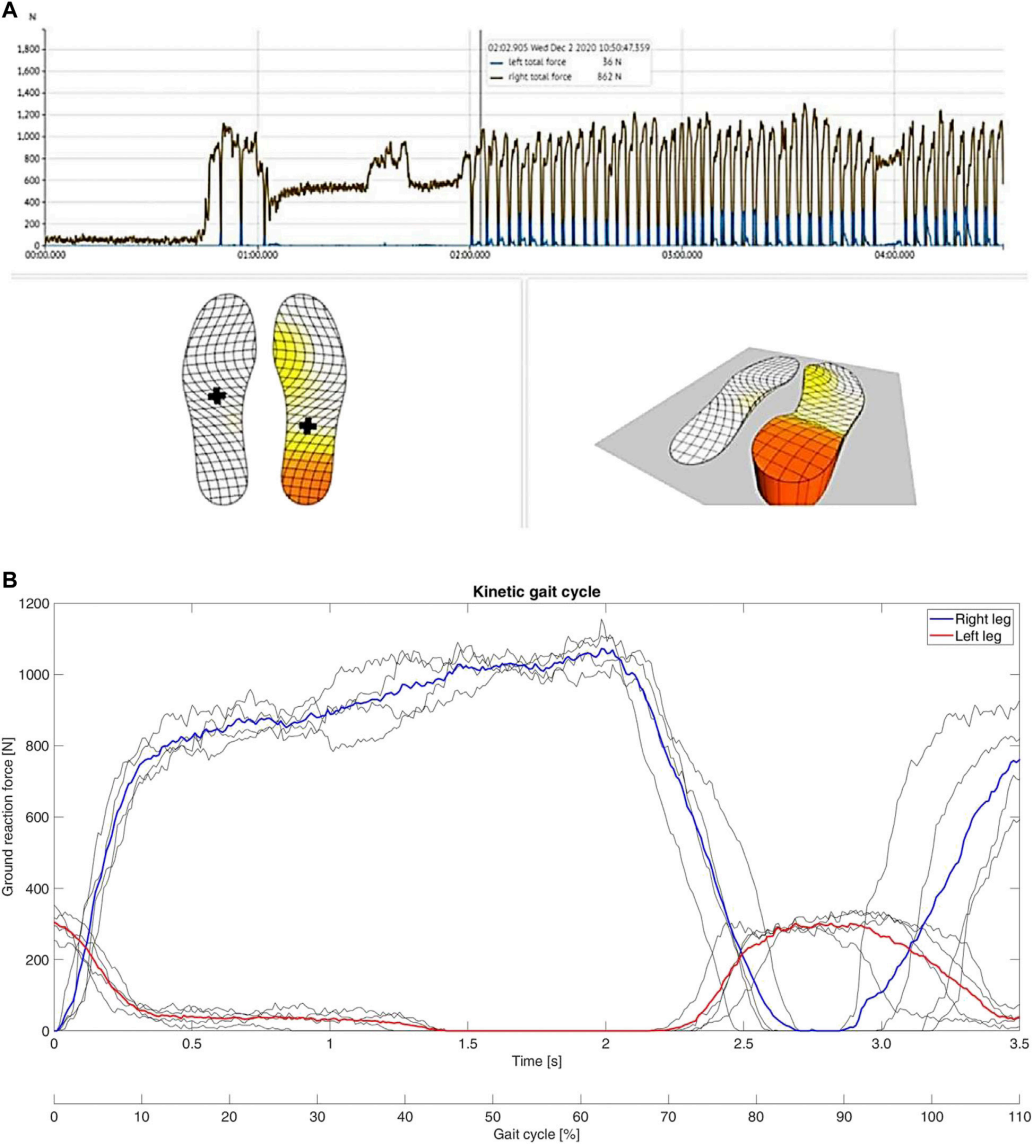
Results of motion data obtained from insoles. **(A)**: Screenshot of the measurement of the insoles shortly after surgery. The right foot is clearly loaded with almost the full weight of the body. **(B)**: Typical curves of the gait cycle during the first physiotherapy session of the patient after surgery. The curves do not show the typical bicuspid shape and the ground reaction force is strongly in favour of the right leg. Of interest, the left leg is loaded with up to approximately 35 kg during the cycles, although weight-bearing restriction of maximum 20 kg was prescribed by the trauma surgeon.

**FIGURE 4:**
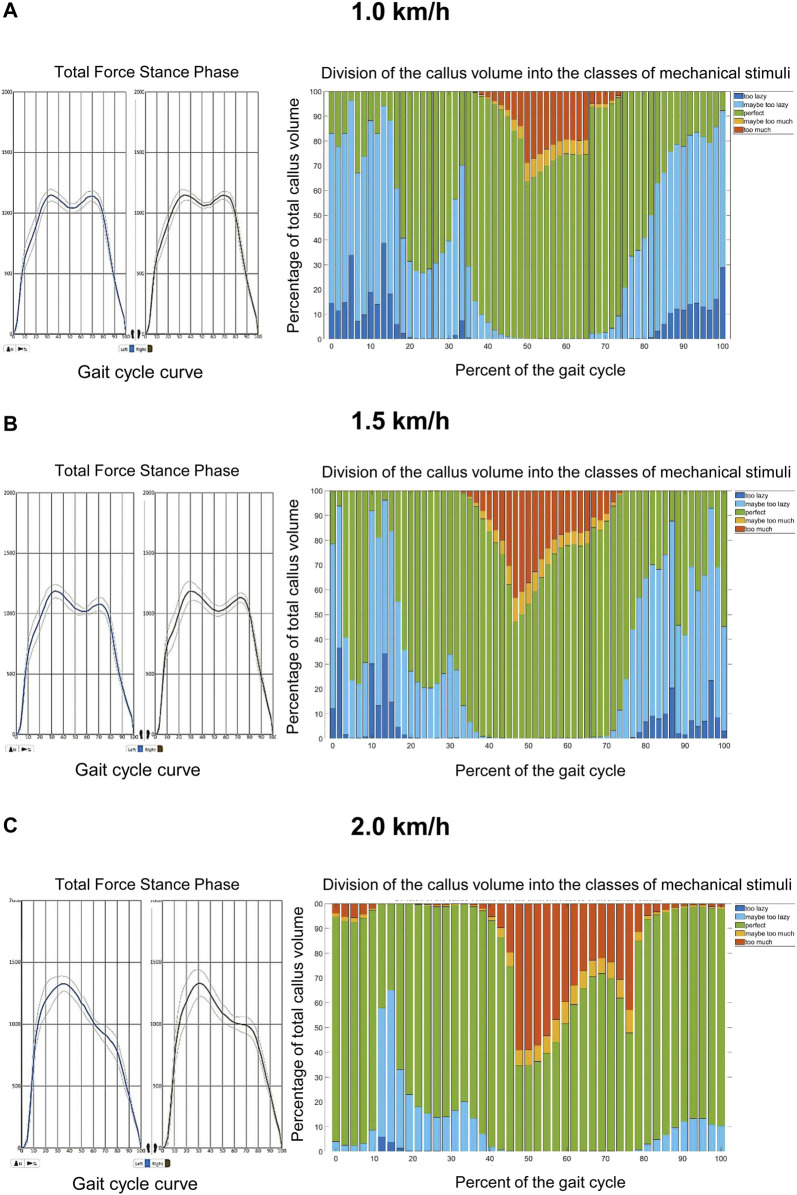
Results of motion data at different walking speeds on the treadmill. **(A)**: At 1.0 km/h the tetrahedral elements located within the fracture gap of the tibia are predominantly within the range of good mechanical properties for healing and bone formation (bars in blue, green and yellow). Only during the middle and end of the stance phase, a short maximum of 38.4% of the elements drop out of this healing range (bars in red). The shape of the gait cycle curve at this velocity has a typical biscuspid form. **(B)**: At 1.5 km/h tetrahedral elements located within the fracture gap of the tibia are also mostly within the range of good mechanical properties for healing and bone formation during the gait cycle (bars in blue, green and yellow). During the middle and end of the stance phase, a maximum of 52.8% of the elements drop out of this healing range (bars in red). The shape of the gait cycle curve at this velocity has a typical biscuspid form. **(C)**: At 2.0 km/h tetrahedral elements out of the range of good mechanical properties for healing and bone formation (bars in red) located within the fracture gap of the tibia can be found throughout the whole gait cycle and show a maximum of 65.3% of all elements during the middle and end of the stance phase, while still most of the elements are in a regular healing window. The shape of the gait cycle curve at this velocity has an atypical monocuspid form.

For further assessment, four models were created from the patient’s lower leg data (Figure 2): 1) Plate osteosynthesis of the distal fibular fracture and intramedullary nailing of the tibia, which resembles the real treatment of the patient (Figure 2A); 2) Untreated fibula (Figure 2B); 3) An intact fibula (Figure 2C) and 4) Removed fibula, only simulation of the treated tibia (Figure 2D).

### 2.3 Acquisition of motion data

Throughout the routine aftercare during the postoperative course, the patient was equipped with a pair of insoles with pressure sensors (Science Insole 3, Moticon ReGo AG, Munich, Germany). The insoles were placed in the shoes of the patient during the first physiotherapy session shortly after surgery. In the lab, the insoles were used to monitor the gait of the patient in a standardized setting of 30 steps on a treadmill (mercury, h/p/cosmos, Nussdorf, Germany) at various velocities (1.0 km/h; 1.5 km/h; 2.0 km/h). For this purpose, the insoles acquired plantar pressure data that were used to compute the resulting average and maximum vertical ground reaction forces and acceleration based on the center of pressure path during the stance phase of gait and its anteroposterior/mediolateral deviation. For every parameter, the mean of all steps and the standard deviation of the measurements between all steps were calculated using the Moticon SCIENCE™ software (Moticon ReGo AG).

Moreover, the patient was monitored *via* an inertial measuring unit (IMU)-based motion capturing system (Xsens MVN Awinda, Xsens Technology B.V., Enschede, Netherlands), as described previously (Braun et al., 2021). Briefly, this system uses seventeen wireless sensors, which are applied to the body of the patient at biomechanically relevant segments. The system measures and processes motion data and provides data in the corresponding evaluation and analysis software Xsens MVN Analyze (Xsens Technology B.V.). This software allows for a comprehensive analysis of the recorded motion data. In addition, the range of motion [flexion (flex)/extension (ex), abduction (abd)/adduction (add), internal rotation (IRO)/external rotation (ERO), and supination (sup)/pronation (pro)] of the individual biomechanical segments of the lower extremities and their joints (hip, knee, ankle joints) was obtained *via* the system. The range of motion in the different joints was compared between the injured leg and the healthy leg of the patient, as well as between different walking velocities.

Finally, the motion capturing data were converted by the MVN software into the Biovision Hierarchy (BVH) data format for export to the musculoskeletal simulation environment AnyBody (AnyBody Technology A/S, Aalborg, Denmark). Together with the collected anthropometric data of the patient, a patient-specific avatar was created in the AnyBody software (AnyBody Technology) that enabled the simulation of individualized muscle forces, ligament forces, and internal joint contact forces and moments, which are essential for the understanding of the biomechanical mechanisms during human movement. These data served as personalized boundary conditions in the different FE simulations and, thereby, enabled the analysis of the effects of walking speed and weight-bearing restrictions on bone healing of the tibia according to the previously described threshold values for the different types of bone healing (Claes and Heigele, 1999; Shefelbine et al., 2005).

### 2.4 Simulation of bone healing after tibial fracture and the effect of the fibula on the healing process

The data from the musculoskeletal simulations were used in conjunction with the generated geometric models to compare the biomechanical scenarios using the software environment Abaqus (Dassault Systemes, Velizy-Villacoublay, France). To investigate the influence of the four different fibular configurations on the von Mises stress distribution of the intramedullary nail, the four models were simulated for high axial loading (midstance of the gait cycle) and high loading with non-axial forces (at the end of the terminal stance of the gait cycle). In this context, von Mises equivalent stress was chosen as scalar quantity representing a measure of local loading which can be interpreted a metric for the distribution of forces. In addition, for these two loading scenarios, the mechanical stimuli and the local micromechanics in the fracture gap of the tibia were investigated with respect to the mechanical conditions for fracture healing. The simulations also allow a comparison between partial and full weight-bearing by adjusting the boundary conditions in each case, which was herein performed for the two load cases of the FibOP model. In addition, the Anybody results were used to simulate complete gait cycles for the three different velocities of the patient on the treadmill. This allowed for an analysis of the fracture healing parameters over the complete gait cycles and, thus, the dynamic influence of gait velocity on the fracture gap and its micromechanics. For this purpose, the hydrostatic strain and the octahedral shear strain were computed for each mesh cell of the callus area, and classified into the different classes with respect to the values given previously by Shefelbine et al., 2005 (Shefelbine et al., 2005). According to this classification, the volumes of the mesh cells were added to calculate the percentages of the total callus volume.

### 2.5 Statistics

All data are given as mean values of all steps ±standard error of the mean (SEM) as well as maximum and minimum values of each step. Data were first tested for normal distribution and the assumption of equal variance was proven. In case of parametric data, comparisons between two experimental groups were performed by an unpaired Student’s t-test, while analyses of three groups were performed by one-way ANOVA, followed by the Holm-Sidak test for all pairwise comparisons, including the correction of the α-error according to Bonferroni probabilities to compensate for multiple comparisons. In case of non-parametric data, comparisons between two experimental groups were performed by a Mann-Whitney Rank Sum Test, while analyses of three groups were performed by one-way ANOVA on Ranks, followed by a Dunn’s Test for all pairwise comparisons, which also included the correction of the α-error according to Bonferroni probabilities. The statistical analyses were performed using the SigmaPlot software 11.0 (Systat Software, Erkrath, Germany). A *p*-value <0.05 was considered to indicate significant differences.

## 3 Results

### 3.1 Clinical results

The clinical course of the patient was uneventful. After intramedullary nailing of the tibia and plate osteosynthesis of the distal fibula, common soft tissue healing occurred. After removal of the set screw approximately 6 weeks after surgery, the patient was allowed to increase load of the injured extremity to full weight-bearing. Due to pre-existing epilepsy, the patient’s gait pattern was already altered before the injury, but full weight-bearing was achieved. Although the tibial fracture showed osseous bridging after 6 weeks, full osseous healing of the tibia was initially delayed especially in the dorsal part of the fracture, but showed full consolidation at 6 months after the surgery. The plate on the distal fibula was removed at approximately 7 months postoperatively after full osseous consolidation. At this stage, the patient was walking free without aids and the fractures of the tibia and distal fibula were fully healed (Figure 1D).

### 3.2 Results of postoperative motion data

Analysis of the data obtained from the insoles demonstrated that the patient avoided full weight-bearing of the injured left leg early after surgery (Figure 3). However, the patient did not adhere to the prescribed maximum weight of 20kg, but instead showed a maximum load of the left leg of approximately 35 kg (Figure 3A). This was recorded even during the first physiotherapy session, although the patient was mobilized under guidance of a physiotherapist (Figure 3B). Analysis of differences in the range of motion of the hip, knee and ankle joints between the injured left and the healthy right side revealed significant differences in all three biomechanical segments and the differences over the segments were seen in all three planes (Table 1). While the left hip showed a reduced flex/ex movement (sagittal plane), the knee showed differences in the rotational degree (axial plane) and the ankle joints had a different extent of sup/pro (frontal plane) (Table 1). Further analyses of the different walking speeds showed velocity-dependent changes in the range of motion (Table 2). Of interest, at 2.0 km/h walking speed the rotation of the injured left knee and the level of abd/add in the right hip and the flexi/ex of the right knee showed significant differences compared to slower walking velocities (Table 2).

**TABLE 1:** Range of motion with global minima (min) and maxima (max) and its mean range (mean∆) of the injured left leg and the healthy right leg over all steps (n = 30) for each joint (hip, knee and ankle joint) and its different directions of movement such as internal rotation (IRO)/external rotation (ERO), flexion (flex)/extension (ex) and supination (sup)/pronation (pro) of the patient on the treadmill independent from the gait velocity. Mean ± SEM; *p < 0.05 vs. left.

	Mean ROM						
		Left (injured)			Right (healthy)		
		min	max	mean∆	min	max	mean∆
Hip	IRO (+)/ERO (-) [°]	3.0	7.8	4.9 ± 0.2	3.3	8.8	4.9 ± 0.2
	Flex (+)/Ex (-) [°]	17.6	22.3	19.5 ± 0.2	14.7	21.8	18.0 ± 0.3*
	Abd (+)/Add (-) [°]	2.0	5.1	3.4 ± 0.2	1.5	5.4	3.2 ± 0.1
Knee	IRO (+)/ERO (-) [°]	1.6	3.2	2.3 ± 0.1	2.4	7.5	3.7 ± 0.2*
	Flex (+)/Ex (-) [°]	32.8	41.3	37.1 ± 0.4	27.9	44.2	36.1 ± 0.6
Ankle	Sup (+)/Pro (-) [°]	4.4	10.3	8.0 ± 0.2	7.1	14.5	10.9 ± 0.3*
	Flex (+)/Ex (-) [°]	7.0	18.2	11.3 ± 0.5	6.7	17.2	11.5 ± 0.5

**TABLE 2:** Range of motion with global minima (min) and maxima (max) and its mean range (mean∆ ± SEM) of the given quantities over all steps (n = 10) for each joint (hip, knee and ankle joint) and its different directions of movement such as internal rotation (IRO)/external rotation (ERO), flexion (flex)/extension (ex) and supination (sup)/pronation (pro) of the patient on the treadmill at different velocities. Mean ± SEM; *p < 0.05 vs. 1.0 km/h; #*p* < 0.05 vs. 1.5 km/h; §*p* < 0.05 vs. 1.0 km/h.

		1.0 km/h						1.5 km/h						2.0 km/h					
		Left			Right			Left			Right			Left			Right		
		min	max	mean∆	min	max	mean∆	min	max	mean∆	min	max	mean∆	min	max	mean∆	min	max	mean∆
Hip	IRO (+)/ERO (-) [°]	−3.2	3.8	4.2 ± 0.3	−0.9	5.5	4.2 ± 0.3	−3.0	4.2	4.6 ± 0.2	0.9	8.5	5.0 ± 0.3	−4.1	4.2	5.9 ± 0.3*#	−0.9	8.8	5.6 ± 0.5*
	Flex (+)/Ex (−) [°]	2.1	22.9	18.9 ± 0.3	2.8	23.2	17.3 ± 0.3	0.7	24.9	19.9 ± 0.4	3.9	26.1	19.5 ± 0.4**§**	6.5	30.0	19.9 ± 0.3	6.9	29.2	17.3 ± 0.5**#**
	Abd (+)/Add (−) [°]	−5.3	−0.7	3.4 ± 0.2	−0.5	4.5	2.9 ± 0.2	−4.4	−0.6	3.9 ± 0.2	0.1	6.0	2.9 ± 0.1	−6.9	−0.8	2.9 ± 0.3**#**	−1.5	3.3	3.8 ± 0.3*#
Knee	IRO (+)/ERO (−) [°]	−1.6	1.7	2.2 ± 0.1	−2.9	1.7	3.1 ± 0.2	−1.5	1.5	2.1 ± 0.1	−2.5	2.6	3.5 ± 0.2	−1.7	1.5	2.6 ± 0.1*#	−4.9	2.6	4.4 ± 0.5*
	Flex (+)/Ex (−) [°]	0.1	38.8	35.0 ± 0.5	0.9	41.8	36.5 ± 0.6	0.4	41.5	38.7 ± 0.4**§**	2.2	46.4	39.0 ± 0.8**§**	2.0	45.1	37.6 ± 0.6*	4.1	46.0	32.8 ± 0.9*#
Ankle	Sup (+)/Pro (−) [°]	−6.5	4.5	7.3 ± 0.5	1.6	17.3	11.2 ± 0.6	−6.9	3.0	7.9 ± 0.3	3.4	18.0	11.5 ± 0.4	−6.8	4.1	9.0 ± 0.3*	1.0	19.6	10.1 ± 0.6
	Flex (+)/Ex (−) [°]	7.5	−5.9	9.7 ± 0.6	9.0	−5.7	10.6 ± 0.6	8.1	−7.8	13.2 ± 0.5**§**	10.2	−7.4	13.6 ± 0.9**§**	6.5	−11.7	10.9 ± 1.1	9.5	−4.4	10.2 ± 0.6**#**

### 3.3 Impact of walking speed on the interfragmentary strain

Walking on the treadmill at different velocities revealed characteristic changes during the gait cycle of the patient (Figure 4). While the ground reaction force was not different between velocities, the shape of the gait cycle ground reaction force curve changed from the typical shape with two maxima at 1.0 and 1.5 km/h (Figures 4A, B to only one maximum at 2.0 km/h (Figure 4C). Moreover, in the fracture gap the number of tetrahedral elements in the range of good mechanical properties for healing and bone formation changed during the gait cycle and showed a speed-dependency. While practically all elements were within the range of good mechanical properties for healing and bone formation at the beginning and end of the gait cycle at 1.0 km/h, this amount dropped to 63.6% of the callus volume during the middle and end of the stance phase (Figure 4A). Of interest, this value dropped further to 47.2% at 1.5 km/h (Figure 4B) and even 34.7% at 2.0 km/h (Figure 4C). Moreover, at 2.0 km/h elements within the fracture gap outside the regular healing range could also be detected at the beginning and at the end of the gait cycle (Figure 4C) and, thereby, demonstrated a potential negative effect of higher walking speed on the bone healing process. These results were also confirmed by dynamic visualization of the elements within the simulated callus at the different velocities (Supplementary Video S1).

### 3.4 Impact of weight-bearing restrictions on the interfragmentary strain in the tibia

**FIGURE 5:**
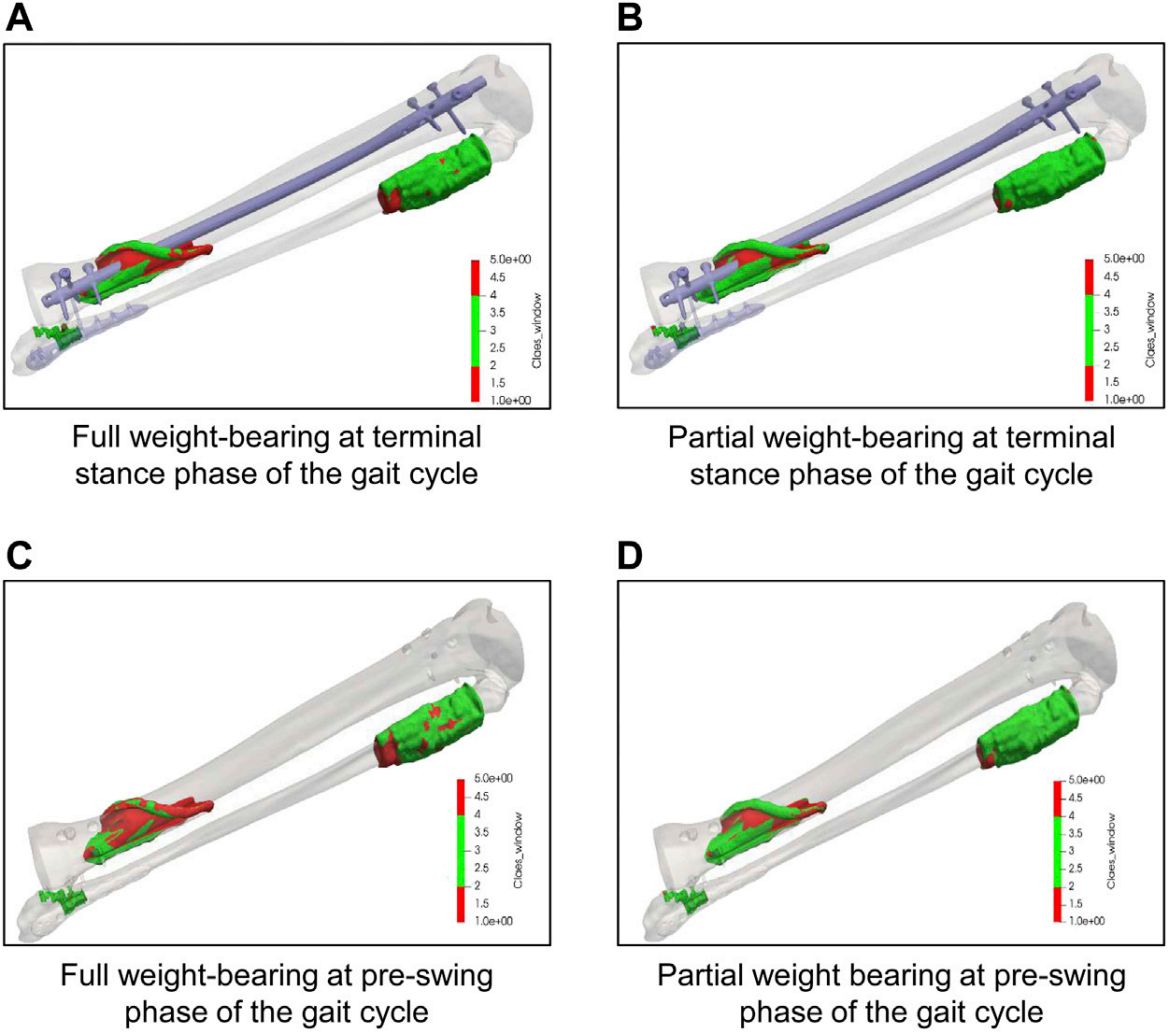
Results of simulation with different weight-bearings on the interfragmentary strain in the tibia. **(A**, **B)**: Simulation of elements within the fracture gap of the tibia at the terminal stance phase during the gait cycle under full weight-bearing **(A)** and partial weight-bearing of 20 kg **(B)** demonstrating the amount and precise location of elements within (green) or outside (red) the range of regular bone healing conditions according to Claes and Heigele (1999). **(C**, **D)**: Simulation of elements within the fracture gap of the tibia at the pre-swing phase during the gait cycle under full weight-bearing **(A)** and partial weight-bearing of 20 kg **(B)** demonstrating the amount and precise location of elements within (green) or outside (red) the range of regular bone healing conditions according to Claes and Heigele (1999). Note that most of the elements out of the healing range were found to be in the dorsal aspect of the tibia fracture, which, in turn, corresponds to the clinical course of events.

Simulation of different weight-bearing restrictions after surgical treatment at the terminal stance phase and the pre-swing phase during the gait cycle revealed that more elements were outside the healing window when the patient was allowed to fully weight-bear compared to a partial weight-bearing restriction of 35 kg (Figure 5). Moreover, the simulations demonstrated more elements outside the healing range in the pre-swing phase than at the terminal stance phase, independent of the loading on the injured leg. Of interest, the geometric model of the lower leg allows not only for a general finding for all elements within the fracture gap, but precisely locates in which area of the fracture gap elements out of the healing range can be found. By these means, most of the elements outside healing range were found in the dorsal aspect of the tibial fracture, which, in turn, corresponds to the clinical course of events, where healing was delayed dorsally.

### 3.5 Impact of the different fibular configurations on the von mises stress in the tibial intramedullary nail

**FIGURE 6:**
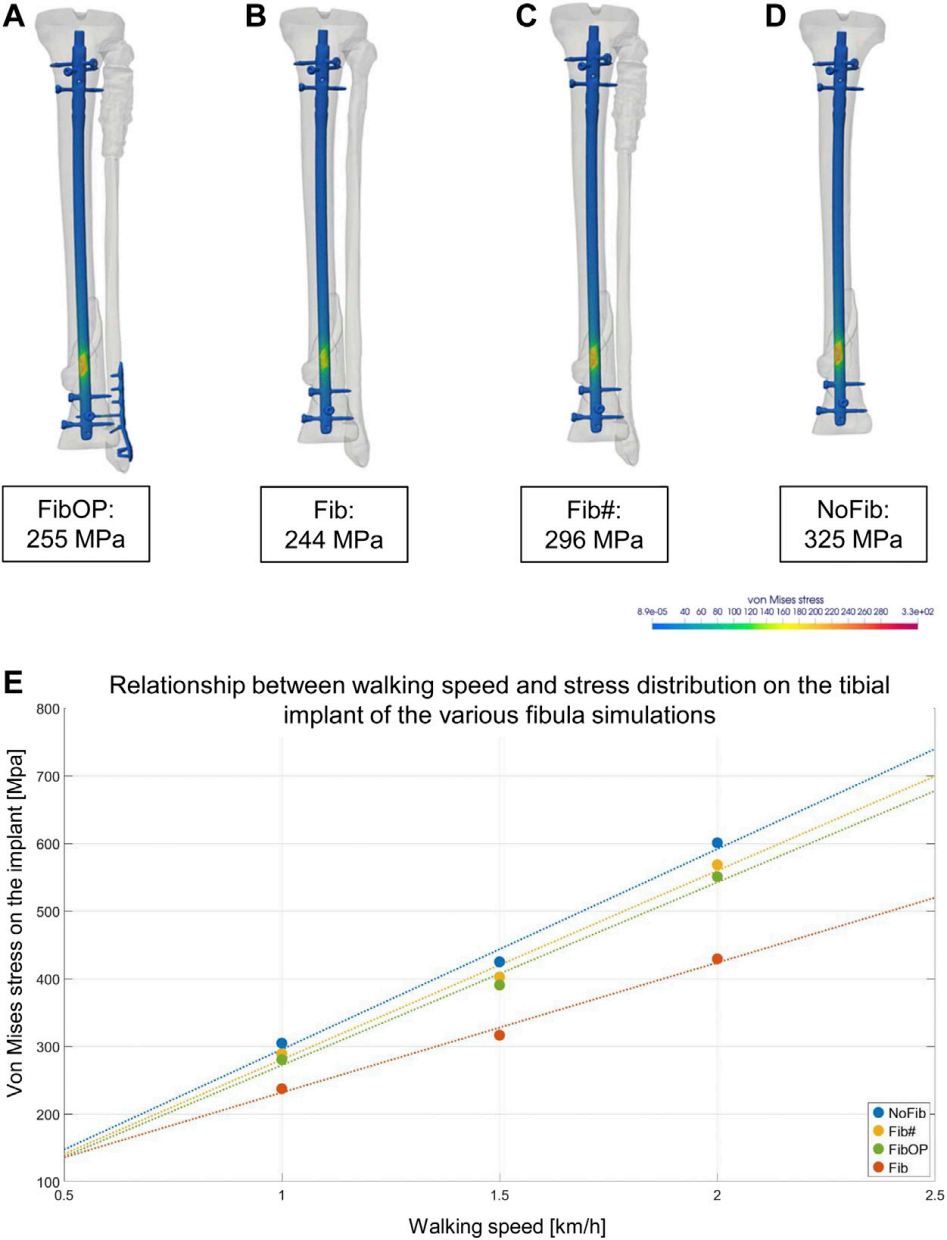
Results of the different FE simulations of the fibula and its impact on the stress maxima on the intramedullary nail. A-D show the von Mises equivalent stress for the moment of maximum axial loading during a patient’s step forward at his first physical therapy session. **(A)**: Simulation of plate osteosynthesis of the distal fibula (FibOP) corresponding with the real treatment. **(B)**: Simulation of unfractured fibula (Fib). Note the only slight difference of the strain maximum to FibOP. **(C)**: Simulation of proximally and distally fractured fibula (Fib#) without surgical intervention. Note the increase of the von Mises stress on the implant. **(D)**: Simulation of excluded fibula (NoFib) with further increase on the intramedullary nail in the tibia, demonstrating the impact of the fibula and its stability on the von Mises stress within the tibia. **(E)**: The various fibula simulations and their impact on the tibial implant in relation to the walking speed of the patient.

The four simulations with different bone and implant configurations of the fibula as described in Figure 2 revealed different von Mises stress maxima on the intramedullary nail surface (Figure 6). The FibOP simulation showed a maximum of 255 MPa on the nail in the area of the fracture (Figure 6A) and a maximum of 83 MPa for the fibular implant. This result was similar to the configuration of an unfractured fibula (244 MPa) (Figure 6B). In contrast, the simulation of no surgical intervention of the distal fibular fracture revealed an increase of the von Mises stress on the intramedullary nail of 16 percent to 296 MPa (Figure 6C). Although the fibula was fractured proximally and distally, a minimal stability remained as it slightly reduced the von Mises stress on the nail in comparison to the configuration NoFib (325 MPa) (Figure 6D). Of interest, the various fibular simulations in relation to the patient’s motion data revealed increased von Mises stress on the implant with increasing walking speed (Figure 6E).

## 4 Discussion

The present study showed that increasing walking speed and loading raised the strain in the fracture gap and in the intramedullary nail, and that the stability of the fibula affects the healing of the tibial injury. Based on the present case, FE simulations showed that fibular fractures should be stabilized surgically when they appear to be on the same level as the tibial fracture, while proximal fibular fractures above tibial injuries do not require surgical treatment. FE simulations further enable patient-specific recommendations in combination with an analysis of the patient’s gait including the range of motion of the lower extremities during the postoperative aftercare, and may, by these means, lead to novel, prognostic individual recommendations.

Surgical treatment of a fibular fracture in combination with an ipsilateral tibial fracture is discussed controversially in the literature. Although additional fibular fixation has shown to improve stability and resistance to torsional forces, there has been increased potential for soft tissue-related complications and therefore the clinical impact has been questioned (Varsalona and Liu 2006; Morin et al., 2008). The biomechanical purposes of the fibula are to stabilize the ankle joint on uneven surfaces and to store elastic energy to jump (Rittweger et al., 2018). Due to these main functions and the fact that it only carries a very small portion of the body weight, other than the tibia, during immobilisation the fibula is less influenced by disuse (Ireland et al., 2017). Even when a large part of the fibula is removed for free fibular osteoseptocutaneous flaps in mandible reconstruction, the gait and lower leg functions are only altered to a small degree (Lin et al., 2009). One might argue that, in line with these facts, there is no need to surgically address the fibula in lower leg fractures when the ankle joint is properly aligned. However, in the presented case, the surgical stabilization of the distal fibular fracture lead to a reduced strain maximum within the tibial fracture. As far as conclusions can be drawn from the herein presented case, this suggests that surgical treatment of the distal fibular fracture is recommendable to facilitate healing. In contrast, the proximal fibular fracture does not seem to influence the strain distribution within the tibial fracture, as the analysis of von Mises stress on the intramedullary tibial nail exhibits almost identical results between the models FibOP and Fib. Taking into account that surgical treatment always bears risks and potential complications as, e.g., damage to the peroneal nerve at the proximal end of the fibula, surgical treatment of the proximal fibula is not recommended in this case. These findings are in line with a number of previous studies analyzing the effect of surgical treatment of the fibula on bone healing of the tibia with concomitant fracture of the fibula in clinical cases (Strauss et al., 2007; Berlusconi et al., 2014).

Lately, it has been shown that the location of the fibular fracture is important to determine whether the fixation is indicated or not. Fibular osteosynthesis was considered advisable in distal metaphyseal fracture of the fibula with trans- or infrasyndesmotic lesion (Pogliacomi et al., 2019). In the present study, we found similar results by simulating the individual injury and its surgical treatment. As demonstrated in Figure 6, this approach allows the analysis of various potential surgical treatment options in consideration of the individual morphology of the patient’s injury. Since medical imaging programs nowadays allow digital reduction of a fracture for preoperative planning, it might even be possible to give a valid, individual prognostic estimation of the healing course for the patient immediately after the surgery. Together with individualized acquisition of motion data in the postoperative course, the simulation itself is validated and enables the treating surgeon to individually track the bone healing process. Thereby, the use of FE simulations as an additional tool for clinical applications may have a great impact on the future treatment of trauma patients and bear advantages in comparison to common retrospective clinical studies.

The early postoperative treatment for trauma patients is commonly performed by general, diagnosis-related recommendations on weight-bearing and the type of mobilization. Radiographic controls are initially performed in intervals of approximately 6 weeks with increasing time spans in the later stages of the aftercare period. By these means, problems and complications such as, e.g., delayed bone healing or non-union formation during that period of treatment are mostly detected after several months, causing an individual and overall increased burden of disease (Ekegren et al., 2018). In order to detect and if possible to prevent these complications, it is necessary to identify prognostic factors that (i) influence the bone healing process, (ii) can easily be monitored especially throughout the early but also the later phases of bone healing and (iii) can easily be controlled by the patient and the treating surgeon. In the present study, we likely identified the two parameters walking speed and weight-bearing restriction to have an impact on the bone healing process *in silico*. While weight-bearing is already widely accepted to be influential for bone healing but is known to have a low compliance rate (Frost, 2004; Ganse et al., 2016; Braun et al., 2017), we showed by the simulation derived from individual motion data, that the different phases and the walking speed of the gait cycle have an impact on the force elements within the fracture gap. During the phases of maximum force in axial direction (terminal stance phase) and maximum of occurring moments (pre-swing phase), and increase of walking speed to 2.0 km/h revealed more elements to be outside the range of beneficial mechanical properties for bone healing and bone formation (Claes and Heigele, 1999; Shefelbine et al., 2005). Moreover, the higher walking speed of 2.0 km/h corresponded to a change of the gait cycle curve (a loss of the two maxima), while the walking speed of 1.5 km/h did not lead to changes of the gait cycle curve and showed only slight changes of elements outside the biomechanical window of bone healing compared to 1.0 km/h (Figure 4). Bending and torsion in the human tibia are known to positively correlate with walking speed (Yang et al., 2014; Yang et al., 2015). Accordingly, it may be speculated that an increased walking speed affects the bone healing and that the effect becomes detrimental when the patient’s curve of the gait cycle individually changes from the typical shape with two maxima to an atypical, e.g., shape with only one maximum. This also corresponded clinically to the differences of the rotation and flex/ex movement of the left knee, as well as the abd/add of the contralateral hip. Based on the fact that elements outside the optimal healing range were found independently from the walking speed in all gait velocities, it may be assumed that despite the duration during the gait cycle, also the volume of elements within the fracture zone may be of pivotal importance for the outcome of bone healing during the overall healing phase. Therefore, future studies are necessary to define the critical volume and lower threshold of the duration for elements within the healing window in order to promote bone healing of a fracture. These parameters may then even be mapped to different zones of the callus to predict healing (Figure 5).

We are aware that these conclusions are drawn from only one case in the present study, and more cases will have to be provided to further analyze these associations. However, for individualized aftercare the herein introduced monitoring of the walking speed in combination with changes of the shape of the gait cycle curve in consideration of potential weight-bearing restrictions, as well as potentially other kinetic and kinematic parameters may be helpful novel tools for clinical follow-up controls of bone healing. In fact, this is in line with previous studies analyzing the relevance of gait patterns on bone healing in an experimental setting (Seebeck et al., 2005; Kröger et al., 2022). Also, it has been demonstrated that the initial phase of healing is sensitive to mechanical conditions and influences the course of healing (Klein et al., 2003). Therefore, monitoring mechanical conditions by gait analysis with respect to threshold values for interfragmentary strain and hydrostatic pressure during the first days after surgery may help to transform general, retrospectively-based recommendations to individual, prospective and radiation-free recommendations on postoperative treatment in the future.

In future finite element analyses, it might be of interest to implement callus growth over time and stiffness changes in the fracture gap throughout the course of healing to study the development of strain and stress maxima over time (Naveiro et al., 2021; Wan et al., 2022). In addition, finite element simulations might be beneficial that include the muscular pull and the influence of soft tissues, as well as calculations of movement in the fracture gap (Yang et al., 2015; Moazen et al., 2019).

## 5 Conclusion

In conclusion, we demonstrated an individualized simulation of a complex fracture of the lower leg based on the patient’s image and motion data. Together with patient-specific early postoperative motion data, the current findings might enable the treating trauma surgeon to estimate individually the healing course within the first days after surgery. Moreover, the impact of walking speed on the mechanical condition monitored by the gait cycle curve during the postoperative course is demonstrated and highlights the need to find thresholds for the duration and the volume of elements in good healing conditions in future studies. Walking speed in combination with changes of the shape of the gait cycle curve and differences of the range of motion in the involved joints may be helpful novel tools for follow-up visits in clinical practice. This may help to develop individual, prospective and radiation-free recommendations on postoperative treatment in the future.

## Data Availability

The original contributions presented in the study are included in the article/Supplementary Material, further inquiries can be directed to the corresponding author.

## Supplementary material

SUPPLEMENTARY VIDEO S1 Dynamic 3D model of the individualized fracture of the lower leg after surgery during a mean gait cycle with a walking speed of 1.0 km/h, 1.5 km/h, and 2.0 km/h. The different colours of elements within the fracture gap of the tibia during the gait cycle represent the division of the callus into the different classes of mechanical stimuli (dark blue: no movement; light blue: insufficient movement; green: perfect healing; yellow: maybe too much movement; red: too much movement). Note the increased amount of red elements during the gait cycle at 2.0 km/h.

SUPPLEMENTARY DATA SHEET S1 Detailed description of the workflow for reproducibility.
