# Supplementary material for: Simulation-based prediction of bone healing and treatment recommendations for lower leg fractures: Effects of motion, weight-bearing and fibular mechanics
Source: Front Bioeng Biotechnol. 2023 Feb 20;11:1067845. doi: 10.3389/fbioe.2023.1067845 (PMC9986461; doi:10.3389/fbioe.2023.1067845)
Supplement: Supplementary file 1 [file DataSheet1.PDF]

Supplementary Material 2

Simulation-based prediction of bone healing and treatment recommendations for lower leg fractures: effects of motion, weight-bearing and fibular mechanics

M. Orth*, B. Ganse, A. Andres, K. Wickert, E. Warmerdam, M. Müller, S. Diebels, M. Roland, T. Pohlemann

*** Correspondence:** Corresponding Author: marcel.orth@uks.eu

1 Supplementary Details on Methodology

The patient's motion data were recorded with the commercial Xsens measurement system, as described in the article. To achieve individualized results in the evaluation, the biomechanically relevant segment lengths were also measured on the patient. The motion data, together with the measured segment lengths, are then transferred to AnyBody using the interface provided by the software. This process has been jointly developed by both companies and is part of the commercially available software. A detailed description is available under the link: https://tutorial.xsens.com/video/importing-into-anybody.

To further customize the avatar in AnyBody to our model patient, we used additional measurements of the patient for a further personalization as described in the AnyBody documentation. The software outputs the joint forces and moments over time in the form of data vectors, which are then used as input data in our FEM software.

In Abaqus (our FEM environment) there is the possibility to create data vectors as so called "Amplitudes". We decided to import the AnyBody output data as “Tabular”-type “Amplitudes” separately for each spatial direction, e.g. knee forces in x-direction etc.

The number of data points then corresponds to the length of the “Time period”, which must be specified in Abaqus in the “Step” definition. We then used the created data vectors as boundary conditions for the simulation. For this purpose, they were stored as "Concentrated force" in the "Load" module of Abaqus. For example, this was implemented for the knee forces in the x-direction as follows: "Distribution" of the type "Uniform" with “CF1” was set to one and “CF2” and “CF3” were set to zero and the “Amplitude” was set to the defined data vector from the “Amplitudes” module as described above. On the foot side, we have chosen to use homogenous Dirichlet boundary conditions for all degrees of freedom. By far the most complex problem is the positioning of the boundary conditions on the biomechanically relevant or correct surfaces and areas on the respective bones. We solved this part outside of the FEM software in our image processing software Simpleware ScanIP by creating the surfaces manually as separate masks and then writing them out as "node sets" directly in the Abaqus input files. Simpleware ScanIP offers this option both for the definition of node sets and for the creation of contact boundary conditions, so that these can be easily retrieved later in FEM programs and used directly. Our workflow is relatively straight forward and should actually be easy to replicate by anyone using the same software chain. Likewise, individual software components can be replaced by other products on the market or in-house solutions. This would lead to the same solution strategy and deliver comparable results.
